# Intracellular adhesion molecule 1 in prodromal Alzheimer’s disease

**DOI:** 10.1002/alz.089716

**Published:** 2025-01-09

**Authors:** Qumars Behfar, Stefan Kambiz Behfar, Oezguer A. Onur

**Affiliations:** ^1^ Department of Neurology, Faculty of Medicine and University Hospital Cologne, University of Cologne, Cologne, Germany, Cologne, north‐rhine westphalia Germany; ^2^ HES‐SO University of Applied Sciences and Arts of Western Switzerland, Geneva, Switzerland, Geneva, Geneva Switzerland; ^3^ Department of Neurology, University of Cologne, Medical Faculty, Cologne Germany

## Abstract

**Background:**

Age represents the predominant risk factor for Alzheimer's disease (AD) dementia. Nevertheless, not every elderly individual undergoes age‐related processes that inevitably lead to dementia. The aging process is characterized by cellular senescence, manifesting as morphological changes and the secretion of immune signaling mediators linked to systemic low‐grade inflammation. Consequently, our investigation aimed to assess the correlation between cerebrospinal fluid (CSF) levels of specific senescence‐associated secretory phenotype (SASP) proteins and accelerated biological aging. Furthermore, we sought to determine whether this acceleration has an impact on the progression along the Alzheimer's disease continuum.

**Method:**

Biological age was computed utilizing established epigenetic clocks, employing genome‐wide DNA methylation (DNAm) data derived from blood samples collected in the Alzheimer’s Disease Neuroimaging Initiative (ADNI). In addition, BrainAge, a neuroimaging‐based aging clock, was calculated. Age acceleration was ascertained through residuals obtained by regressing predicted biological age against chronological age. Linear and Cox regressions were employed to evaluate the correlation between SASP and the acceleration of biological age, as well as the associated risk of progression from prodromal mild cognitive impairment (MCI) to AD.

**Result:**

In our SASP panel, intercellular adhesion molecule 1 (ICAM‐1), Interleukin 8 (IL‐8), matrix metalloproteinase 3 (MMP‐3), plasminogen activator inhibitor 1 (PAI‐1), tissue inhibitor of metalloproteinases 1(TIMP‐1), vascular endothelial growth factor (VEGF) and stem cell factor (SCF) associated significantly with chronological age. Next, we observed that DNAm acceleration was associated with higher risk of progression to AD, and ICAM‐1 was the only SASP factor which was found associated with DNAm age acceleration, and explained part of the effect of age‐acceleration on progression from MCI to AD.

**Conclusion:**

Our findings provide supporting evidence on the contribution of biological age‐acceleration to the progression of AD. Herein, ICAM‐1 might serve as a standalone biomarker determining how biological age‐acceleration affects the progression along the AD continuum.

